# The Influence of Diet and Obesity on Gene Expression in SLE

**DOI:** 10.3390/genes10050405

**Published:** 2019-05-27

**Authors:** Antonio La Cava

**Affiliations:** Department of Medicine, University of California Los Angeles, 1000 Veteran Ave. 32-59, Los Angeles, CA 90095-1670, USA; alacava@mednet.ucla.edu; Tel.: +1-310-267-4975; Fax: +1-310-206-8606

**Keywords:** diet, obesity, systemic lupus erythematosus

## Abstract

This review provides an overview of the known effects of diet, obesity, and the intake of different nutrients on systemic lupus erythematosus (SLE). It summarizes and discusses the studies in rodents that identified how different diets can regulate gene expression in the disease, together with a description of the effects of diet on lupus patients’ inflammatory state and disease severity. The identification of selected dietary candidates that can modulate SLE onset and progression is analyzed in relation to possible targeted approaches that could ultimately ameliorate the management and prognosis of this disease.

## 1. Introduction

Systemic lupus erythematosus (SLE) is a systemic autoimmune disease characterized by immune responses to multiple self-antigens, mostly of intracellular origin and including cell nucleus and DNA molecules. The dysregulation of innate and adaptive immune responses in SLE is secondary to several abnormalities that include an impaired clearance of apoptotic cells and an increased availability of self-antigens that induce production of autoantibodies that can form immune complexes with their cognate antigen, fix complement, and deposit in tissue where they can cause damage and impaired organ function [1].

Genetics alone is not sufficient to explain the development of SLE, and environmental triggers influence the development and expression of the disease. Among the environmental factors that modulate the onset and progression of SLE, diet plays a critical role. Nutrients not only impact overall health but also alter the gut microbiota composition and the immune response. In other words, the host’s metabolic status, which highly depends on diet, can favor or not the development of immune abnormalities manifesting with varied symptoms and/or organ involvement that depend on the genetic backgrounds and additional factors [2]. This explains why patients with SLE often benefit from individualized dietary changes tailored according to their condition, in addition to general approaches of weight loss when obese.

## 2. Diet and Gene Expression in SLE

### 2.1. PUFA

Both lupus-prone mice and patients with SLE benefit from a dietary consumption of n-3 polyunsaturated fatty acids (n-3 PUFA). Mammals, including humans, are not able to synthesize PUFA and thus require those nutrients from exogenous sources such as fish oil [3]. PUFA are found in dietary oils from plants such as corn and soybean, where linoleic acid is most represented [4].

The beneficial effects of dietary n-3 PUFA have been associated with anti-inflammatory activities induced by the suppression of proinflammatory cytokine production and the promotion of antioxidant enzyme activities, in addition to a reduced immune effector activity and a decreased antigen presentation [5].

Clinical trials have provided evidence that the consumption of fish oil benefits lupus patients [6,7]. Those trials followed encouraging studies in mice that had indicated that a diet that included fish oil provided beneficial effects in multiple strains of lupus-prone animals. In addition to delaying lupus disease manifestations, fish oil reduced the expression of IL-1β, TNF-α, and ICAM-1 in (NZB x NZW)F_1_ (BW) mice, while it increased the expression of antioxidant enzymes [8,9]. Reduced lupus disease manifestations were seen, as well, in BXSB/MpJ and MRL-lpr lupus-prone mice [10], together with a reduced expression of the proinflammatory cytokines IL-6, IL-12, and TNF-α [11]. Further comparisons among different dietary lipids confirmed the beneficial effects of fish oil on lupus manifestations in BW mice. In those experiments, one group of BW mice received a diet rich in PUFA with docosahexaenoic acid-enriched fish oil (DFO). A second group received n-6 PUFA-rich western-type diet, containing corn oil (CRN), and the third group received n-9 monounsaturated fatty acid (MUFA)-rich Mediterranean-type diet containing high oleic safflower oil (HOS). Mice fed n-6 PUFA or n-9 MUFA developed clinical manifestations of SLE including autoantibodies and renal disease, while mice fed n-3 PUFA diet did not develop similar signs [3] (yet other studies had found protective effects deriving from n-6 PUFA [12]). Gene expression analyses on sorted CD4^+^ T cells, the helper cells in the production of autoantibodies from B cells, provided a comparison, by PCR array, of the relative expression of 84 genes in kidneys and spleens from the three different groups of mice. At week 34, 33 genes were downregulated and 3 upregulated in kidneys of DFO-fed mice as compared to HOS-fed mice, and 37 were downregulated and 2 upregulated as compared to CRN-fed mice. In the spleens, 26 genes were downregulated and 1 was upregulated in DFO-fed mice as compared to HOS-fed mice, and 25 genes were downregulated and 7 upregulated as compared to CRN-fed mice [3]. The genes whose expression was modulated by DFO associated with inflammatory responses, T and B cell activation/differentiation, leukocyte recruitment, and antigen presentation. Quantitative RT-PCR of representative genes showed that dietary n-3 PUFA associated with a reduced expression of multiple genes involved in proinflammatory responses leading to a reduced expression of IL-6, IL-18 and TNF-α, and reduced expression of CCL-5 chemokine and CXCR3 chemokine receptor, osteopontin, and the costimulatory molecules CD80 and CTLA-4 [3].

### 2.2. Polyphenols

The phenolic fraction of extra virgin olive oil has anti-inflammatory and immunomodulatory properties that associated with a reduced production of pro-inflammatory TNF-α, IL-6, IL-1β, and IFN-γ in 38 lupus patients’ peripheral blood mononuclear cells (PBMCs) [13]. In addition to a reduced T cell (CD69^+^) activation, the phenolic fraction of extra virgin olive oil increased the expression of IκBα due to the prevention of its degradation, leading to downregulation of NFκB (IκBα inhibits NFκB), together with the inhibition of ERK1/2 phosphorylation [13].

### 2.3. Sodium Chloride (NaCl)/Salt Intake

Other studies found that high salt intake in the diet of lupus-prone MRL/lpr mice led to an increased frequency of proinflammatory Th17 and Th1 cells that associated with an increased disease severity and shortened lifespan [14]. Interestingly, the promoting proinflammatory effects of high salt treatment could be reversed in vitro on lupus patients’ Th17 cells co-cultured with serum and glucocorticoid-inducible serine/threonine protein kinase 1 (SGK1), which governs cellular Na^+^ transport and NaCl homeostasis [14].

The deleterious effects of high salt in MRL/lpr mice on the acceleration and worsening of lupus disease manifestations associated with high salt-induced promotion of T follicular helper (Tfh) cell differentiation [15]. Tfh cells have a critical role in the pathogenesis of SLE since they regulate the selection and survival of B cells in the germinal centers, thus contributing to the regulation of autoantibody production in the disease. NaCl-treated CD4^+^ T cells had DNA hypomethylation and increased DNA hydroxymethylation that correlated with higher levels of the hydroxyltransferases ten-eleven translocation (TET)3 and TET2, the latter playing an important role in Tfh cell differentiation (and NaCl-induced Tfh cell promotion via regulation of *Spn* gene expression). In addition to SPN, NaCl increased the expression of SH2D1A, MAP3K1, and STAT5B in SLE patients as compared with controls [15]. By enhancing the expression of MAP3K1 and SPN and by inducing DNA hypomethylation of genes involved in immune responses, NaCl operated at different levels in inducing Tfh cells. During Tfh differentiation, the expression of SPN and STAT5B was upregulated, even more by NaCl, indicating an influence of NaCl on genes involved in Tfh differentiation. Moreover, ChIP-qPCR was performed to further explore the interaction between TET2 and NaCl-increased genes expression. There was TET2 enrichment on *Spn* and *Stat5b*, indicating that increased expression of SPN and STAT5B might associate with enrichment of TET2 after NaCl addition. Gene silencing confirmed that regulation of Tfh cell differentiation by *Spn* was a target of NaCl [15]. Maps of gene expression and DNA methylation of NaCl-treated human CD4^+^ T cells showed that NaCl induced DNA hypomethylation of genes involved in cell differentiation and development (DNA hypomethylation facilitates gene transcription). High salt-treated cells displayed decreased the expression of BLIMP (which promotes Th17, Th1, and Th2 cells) and Th1 (IFN-γ) and Th2 (IL-4 and IL-10) cytokines. The genes that were increased were instead involved in T cell differentiation and activation (*Icos*, *Pdcd1*, *Stat1,* and *Sh2d1a*), including the master regulator of the germinal center reaction *Bcl-6*.

Together, these studies show that high-salt diet could provide an epigenetic contribution to the pathogenesis of SLE.

### 2.4. Vitamins

Dietary supplementation with selected vitamins can have beneficial effects in SLE [16]. Low levels of vitamin D that are common in lupus patients, secondary to the avoidance to sun exposure and/or a reduced dietary intake, have been associated with an increased disease activity. Beneficial effects deriving from vitamin D supplementation in SLE associate with increased numbers of T regulatory cells (Tregs) [17] and the inhibition of the formation of neutrophil extracellular trap (NET)s that cause endothelial damage [18].

Additionally, the administration of the active metabolite of vitamin A, the all-trans-retinoic acid (tRA), also ameliorates SLE in both patients and mice [19,20,21].

### 2.5. Amino Acids and mTOR

A limited consumption of phenylalanine and tyrosine in lupus-prone mice or a moderate protein intake is useful in improving renal function in SLE patients [22]. Accumulation of the tryptophan metabolite kynurenine or the depletion of cysteine and glutathione in SLE promote the activation of the mTOR kinase that fuels inflammation [23,24]. Treatment of lupus patients with the mTOR blockers acetylcysteine [25] or rapamycin [26] provides significant beneficial therapeutic effects that envision potential additional approaches of mTOR blockade (e.g., through calorie restriction [27]) for a possible reduction of the chronic inflammatory state in SLE patients.

## 3. Epigenetic Effects of Diet on SLE

Epigenetics is the branch of biology that studies the stable, reversible, and heritable changes in gene expression resulting from DNA or chromatin modification or post-transcriptional mechanisms that do not associated with changes in DNA coding sequences [28]. These changes include DNA methylation, histone modifications, non-coding RNA expression—i.e. microRNA (miRNA)s, chromatin remodeling, and gene imprinting. Failure to maintain epigenetic homeostasis, e.g. due to environmental causes that can include diet, may result in altered activities at the levels of the cell nucleus, transcriptome, or gene expression.

Diet is a key environmental component that influences host’s gene expression. Altered DNA methylation pattern and subsequent changes in gene expression have been observed in embryos from pregnant mice fed diets that were rich in methyl donors [29]. Dietary supplementation with methyl donors increased DNA methylation in leucocytes [30] whereas a limited dietary intake of methyl donors associated with DNA hypomethylation [31]. This led to the suggestion that diets rich in methyl group donors and essential fatty acids could have beneficial effects on SLE. Metabolomic analyses found that the reduction in energy biogenesis in the disease significantly decreased glycolysis, Krebs cycle, fatty acid β-oxidation, and amino acid metabolism associated with deficiencies of many anti-oxidants including glutathione and methyl group donors [32]. It also elevated markers of oxidative stress from lipid peroxidation products including malondialdehyde, γ-glutamyl peptides and γ-glutamyl transaminase, leukotriene B4, and 5-hydroxyeicosatetraenoic acid [32]. These findings suggested the possibility that these abnormalities could be corrected by dietary intervention [32], and dietary micronutrients necessary for transmethylation were tested in mice to see whether they could influence SLE (lupus patients have a deficiency of methylation-associated micronutrients) [32]. Transmethylation micronutrient–restricted (MR) and transmethylation micronutrient–supplemented (MS) diets were evaluated on the expression of methylation-sensitive T cell genes and lupus disease as mice fed on a low-methionine formulation or a diet rich in methyl donors and cofactorszinc, folic acid, and vitamin B12 [33]. The study found that the diet with reduced methionine content increased lupus disease severity, whereas the diet enriched in methyl donors and cofactors reduced anti-DNA antibody levels and renal disease [33]. Thus, SLE is modulated by DNA methylation that is influenced by the diet, and the interaction of genes and dietary micronutrients can affect murine lupus disease activity and severity.

In addition to methylation, other epigenetic components under investigation in SLE that are influenced by diet are the miRNAs (discussed elsewhere) [34].

## 4. Diet and Microbiome in SLE

Diets can also influence the host’s gut microbiome, imparting changes in the flora composition that have been associated to the pathogenesis of autoimmune diseases [35]. In SLE, the intake of polyphenols influences the composition of the fecal microbiota, and foods rich in flavonoids contribute to an increase in fecal levels of beneficial microorganisms (*Lactobacillus* and *Bifidobacterium*) in lupus patients [36]. On the other hand, interactions between the microbiome and innate and adaptive immune cells can influence immune responses at many levels that include commensal-mediated post-translational modification of self-antigens or the cross-reactivity between commensal and host antigens [37].

## 5. Caloric Intake, Obesity and Gene Expression in SLE

Diets directly influence body weight. Food restriction and/or calorie restriction have long been known to be beneficial in reducing disease manifestations in lupus-prone mice [38,39], including a lowered atherogenic risk, through mechanisms that include a reduced expression of IL-12 and IFN-γ, reduced NF-κB activation [38,40], and the induction of immunosuppressive CD4^+^ Tregs [39].

Although the therapeutic management of SLE that frequently includes the use of corticosteroids affects the patient’s body mass significantly, an excess weight in lupus patients is problematic not only because of its association with impaired functional capacity and fatigue but also because of its implication in worsening the patient’s clinical picture and increased generalized inflammation.

Measurements of the body mass index of lupus patients indicated that about 35% of SLE patients are overweight and about 27% are obese [41]. Another study found that 28% of lupus patients are overweight and 39% are obese, and carriers of higher concentrations of inflammatory markers including C-reactive protein (CRP) [42].

Obese lupus patients have increased gene and protein expression of several proinflammatory molecules such as the cytokines IL-23 [43] and TNF-α, the latter being associated with total fat mass on the trunk region in pediatric lupus patients [44]. This state of inflammation that is associated with obesity creates a background with an increased likelihood to develop complications such as the metabolic syndrome (increased blood pressure, high blood glucose levels, and dyslipidemia). Therefore, obese lupus patients have a high prevalence of metabolic syndrome that directly contributes to the worsening of their chronic inflammatory status because to increased oxidative stress. Together with serum uric acid, increased in the metabolic syndrome, secondary inflammation in obese lupus patients sustains a vicious cycle of oxidative stress through increased CRP gene expression and elevated protein oxidation and lipid hydroperoxidation [45].

## 6. Conclusions

It has become clear over the years that lupus disease activity and severity can be modulated by dietary intervention, as summarized in Table 1.

However, since there are more than 100 metabolites that are altered in lupus patients [32], dietary targeting would need to be as comprehensive as possible and be combined with standard of care. Moreover, a higher success rate may be expected when simultaneously targeting multiple factors or pathways with a relevance in the disease pathogenesis.

Studies in rodents where dietary regimens can be tightly controlled have been informative yet have at times resulted in pitfalls when attempting to extrapolate those results to SLE patients. Notwithstanding these considerations, some dietary approaches that target a specific metabolic pathway with a critical role in lupus pathogenesis, such as transmethylation, have shown promise and are moving from preclinical work toward possible clinical use [33]. Further studies of selected dietary modifications are poised to ultimately lead to improved lupus management, particularly when derived from rigorous prospective and randomized trials [46].

## Figures and Tables

**Table 1:** Dietary components with possible impact on SLE.

Dietary Component	Model System	Target Gene/Protein	Physiopathological Process	Effects	Reference
n-3 PUFA	SLE patients	↓ LTB_4_, IL-12, VLDL	↑ endothelial function, ↓ESR	↓ SLEDAI	[7]
	BW mice	↓ IL-6, IL-12, IL-18, TNF-α, IL-1β	↓ inflammation	↓ lupus nephritis	[8,10]
		↑ catalase, SOD, GPx	↑ anti-oxidant enzymes		
	MRL/lpr mice	↓ LTB_4_, IL-12, PGE_2_	↓ inflammation	↓ disease	[10]
Extra virgin olive oil	SLE patients’ PBMC	↓ IL-6, TNF-α, IL-1β, IFN-γ	↓ inflammation	immune modulation	[13]
NaCl	SLE patients	SPN, SH21A, TET2, TET3, MAP3K1, STAT5B	↑ Tfh cells, DNA hypomethylation	↑ inflammation	[15]
	MRL/lpr mice	SGK1	↑ Th1, Th17 cells	↓ lifespan. ↑ disease	[14]
Vitamin A	SLE patients		↓ inflammation	↓ proteinuria	[19]
	BW mice	↓ IL-2, IL-12, IFN-γ	↓ iNOS, MCP-1	↓ proteinuria, ↑ lifespan	[20]
	MRL/lpr mice	TGF-β	modulation of cytokine expression, apoptosis	↓ lupus nephritis	[21]
Vitamin D	SLE patients	MPO	NETosis	↓ endothelial damage	[18]
Phenylalanine, Tyrosine	BW mice	mTOR	secondary to amino acid metabolites	↓ lifespan. ↑ anti-DNA Ab	[22,23]
Methionine	C57BL/6 × SJL mice	LTB_4_, γ-GT, glutathione	transmethylation	↓ anti-DNA Ab, nephritis	[33]

Ab, antibodies; ESR, erythrocyte sedimentation rate; GPx, glutathione peroxidase; γ-GT, γ-glutamil transaminase; iNOS, inducible nitric oxide synthase; LTB_4_, leukotrien B_4_; MCP-1, monocyte chemoattractant protein-1; MPO, myeloperoxidase; PGE_2_, prostaglandin E_2_; SGK1, glucocorticoid-inducible serine/threonine protein kinase 1; SLEDAI, SLE disease activity index; SOD, superoxide dismutase; TET, ten eleven translocation; Tfh, T follicular helper; Tregs, T regulatory cells; VLDL, very-low-density lipoproteins.
